# Supplementary material for: Preliminary efficacy of a community health worker homebased intervention for the control and management of hypertension in Kiambu County, Kenya- a randomized control trial
Source: PLoS One. 2024 Aug 29;19(8):e0293791. doi: 10.1371/journal.pone.0293791 (PMC11361652; doi:10.1371/journal.pone.0293791)
Supplement: S1 Table — (DOCX) [file pone.0293791.s001.docx]

**S3 Table: Effect of the intervention on Mean changes in blood pressure and body composition measures after 6 months of follow-up**

| **Variable** | **Arm** | Unadjusted mean change between the arms **(95% CI)** | Unadjusted mean change between the arms**(95% CI)** | **P Value** | **Adjusted net mean change**  **(95% CI)** | **P value** |
| --- | --- | --- | --- | --- | --- | --- |
| SBP  (mmHg) | Usual Care | -7.9(-11.8 to - 4.0 ) | -11.1 (-15.2 to -6.9) | <0.001 | -12.4(-16.2 to -8.5) | <0.001 |
|  | Intervention grp | -19(21.04 to -16.9) |  |  |  |  |
| DBP  (mmHg) | Usual Care | -3.8 (-8.5to -0.9) | -6.1 ( -9.2 to -3.0) | <0.001 | -7.8 (-9.8 to -5.7) | <0.001 |
|  | Intervention grp | -9.9(-13.1 to -6.6) |  |  |  |  |
| BMI  (kg/m^2^) | Usual Care | 0.40(-0.002- 0.81) | -1.3 (-1.8 to -0.72) | <0.001 | -1.4(-1.9 to -0.99) | <0.001 |
|  | Intervention grp | -0.90(-1.3 to -0.49) |  |  |  |  |
| WHtR | Usual Care | 0.003(-0.01 to 0.01) | -0.02 (-0.04 to -0.01) | 0.008 | -0.02(-0.04 to -0.01) | 0.007 |
|  | Intervention grp | -0.02(-0.03 to -0.01) |  |  |  |  |

SBP- Systolic blood pressure DBP- Diastolic blood pressure BMI- Body mass index WHtR- Waist height ratio.

For change values, negative number demonstrates improvement. Adjusted analysis was conducted using mixed effects linear regression adjusted for age, gender, BP at baseline, use of antihypertensive medications, BMI and WHtR at baseline.
