# Supplementary material for: Preliminary efficacy of a community health worker homebased intervention for the control and management of hypertension in Kiambu County, Kenya- a randomized control trial
Source: PLoS One. 2024 Aug 29;19(8):e0293791. doi: 10.1371/journal.pone.0293791 (PMC11361652; doi:10.1371/journal.pone.0293791)

**EDUCATION CHECKLIST**

**LIFESTYLE INTERVENTION FOR LOWERING BLOOD PRESSURE**

**Is the client on hypertensive medication?**

If yes inquire on adherence to medication. Encourage the client to show you his/her medication let the client explain how they are taking the medication. Count the pills/tablets from the day of issue to see clients has been taking the medication. Check on the next clinic visit and ensure the medication are adequate to last until the next visit. Advise the client accordingly on need to take the medication as per the doctor’s prescription. In case, of stock-out document and refer to the facility and inform the study coordinator for follow-up to ensure client get te required medication.

**Does the client smoke?**

If yes, give the patient advice on cessation of smoking. Assess for addiction

and dependence such as the last time of smoking and development of withdrawal symptoms.

Offer psychosocial counselling and other support services such as referral to a health facility.

**Does the client take Alcohol?**

If yes, advise the patient to stop taking alcohol or minimize alcohol intake to a maximum of two standard drinks for the men and one standard drink for women. The client should have two alcohol free days per week.

**Body Weight**

Advise the patient with hypertension on how to achieve and maintain a normal body weight with a normal body mass index. The target for a normal weight includes a waist circumference of less than 94cm for men and less than 80 cm for ladies. The normal BMI should be maintained between 19.5-24.5 kg/m 2.

***Consider clients waist circumference and BMI and allow them set monthly targets to be achieved.***

**Physical Activity**

The patient should aim at becoming physically active. The target should be 30 minutes for a moderate intensity activity for at least 5 days a week. Or at least 15 minutes each day for vigorous activity for at least 5 days a week. See examples below. The activities can be classified in short bouts. Advise against heavy exercises that may increase the blood pressure such as lifting weights unless they are supervised.

**MODERATE Intensity Activities: Make you breathe somewhat harder than normal**

- Cycling, Jogging, Brisk walking, Dancing, Horse-riding, Yoga, Low- aerobics
- Cleaning (vacuuming, mopping, polishing, scrubbing, sweeping, ironing), Washing (by hand), Gardening, Milking cows (by hand), Planting and harvesting crops, Digging dry soil (with spade), Weaving , Woodwork e.t.c.

**VIGOROUS Intensity Activities:** Make you breathe much harder than normal

- Soccer, Rugby, Tennis, High-impact aerobics, Aqua aerobics, Dancing, fast swimming
- Sawing hardwood, Ploughing, Cutting crops (sugar cane), Gardening (digging), shoveling sand, Instructing aerobics, masonery.

***Encourage client to maintain weekly log of time spent on physical activity***

***( to be reviewed on monthly basis)***

**Nutrition**

Advice the patient on salt limitation to 4g/day (65mmol/day of sodium) or foods without processed salt. Foods with high salt content such as sausages, ham, canned foods, salty snacks, salt added at the table should be avoided.

**Advice on Dietary Approaches to Stop Hypertension (DASH)- DASH diet**

The patient should be advised to eat a diet which is mainly plant based such as vegetables, pulses, fruits and whole grains.

Additionally, advice the patient to take a low fat or reduced fat dairy products. Little amount of a low-fat dairy products can also be consumed.

Moderate amount of unprocessed meat, fish and poultry are also important into the body. Advice the patients on monounsaturated and polysaturated fats on olive oil, reduced salt margarines.


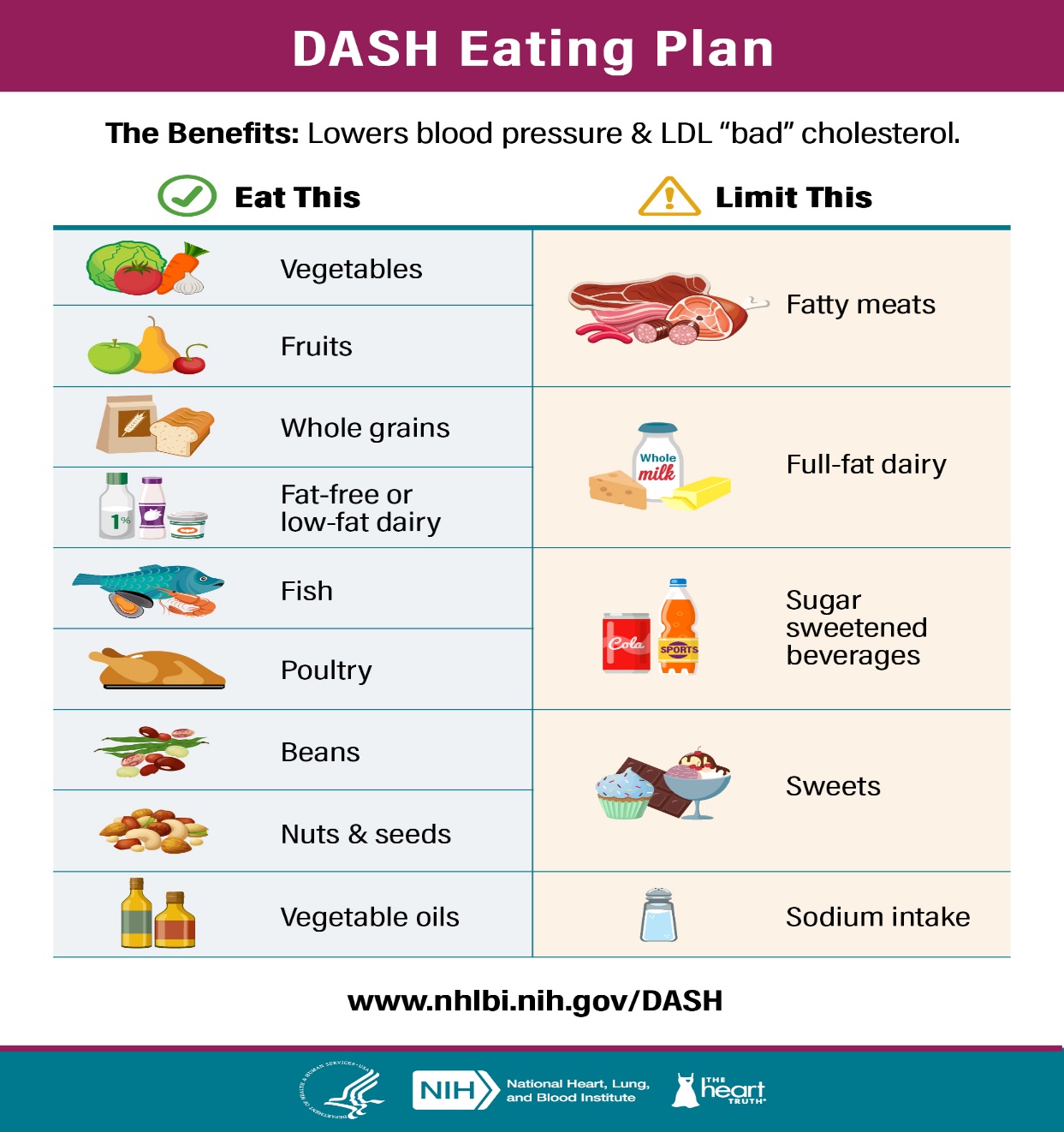

Supplement: S1 File — (DOCX) [file pone.0293791.s002.docx]
