# Supplementary material for: Preliminary efficacy of a community health worker homebased intervention for the control and management of hypertension in Kiambu County, Kenya- a randomized control trial
Source: PLoS One. 2024 Aug 29;19(8):e0293791. doi: 10.1371/journal.pone.0293791 (PMC11361652; doi:10.1371/journal.pone.0293791)
Supplement: S2 File — (DOCX) [file pone.0293791.s003.docx]

**Feasibility and preliminary efficacy of a homebased intervention for the control and management of hypertension amidst the COVID-19 Pandemic in Kiambu County, Kenya**

**Principal Investigator: GRACE W. MBUTHIA Ph.D., MPH. BScN**

**Jomo Kenyatta University of Agriculture and Technology**

**Date: 26^th^ March 2022**

Table of Contents

[Abbreviations/Acronyms 3](#_Toc104455500)

[Abstract 4](#_Toc104455501)

[1.0 BACKGROUND INFORMATION 5](#_Toc104455502)

[1.2 Significance 7](#_Toc104455503)

[1.3 Conceptual framework 8](#_Toc104455504)

[2.0 RESEARCH QUESTIONS AND STUDY OBJECTIVES 9](#_Toc104455505)

[2.1 Research Questions 9](#_Toc104455506)

[2.2 Broad Objectives: 9](#_Toc104455507)

[2.3 Specific Objectives 9](#_Toc104455508)

[3.0 METHODOLOGY 10](#_Toc104455509)

[3.1 Research design 10](#_Toc104455510)

[3.2 Study area 10](#_Toc104455511)

[3.3 Study population 10](#_Toc104455512)

[3.3.1 Inclusion criteria 10](#_Toc104455513)

[3.3.2 Exclusion criteria 10](#_Toc104455514)

[3.4 Sample size and power calculation 10](#_Toc104455515)

[3.5 Sampling and participants recruitment 10](#_Toc104455516)

[3.6 Data collection 11](#_Toc104455517)

[3.7 Data for feasibility and acceptability assessment 12](#_Toc104455518)

[3.8 Study procedure 12](#_Toc104455519)

[3.9 Data analysis 13](#_Toc104455520)

[3.10 Ethical considerations 13](#_Toc104455521)

[REFERENCES 15](#_Toc104455524)

[APPENDIX 1: SURVEY QUESTIONNAIRE 20](#_Toc104455526)

[An adapted WHO stepwise questionnaire to collect prevalence of cardiometabolic risk factors among low income population in Kiambu County. 20](#_Toc104455527)

[APPENDIX 3: CLIENT SATISFACTION QUESTIONNAIRE. 24](#_Toc104455528)

#

# Abbreviations/Acronyms

BP: Blood pressure

CMRFs: Cardiometabolic risk factors

CHW: Community health workers

CVD: Cardiovascular diseases

DASH: Dietary approaches to stop hypertension

IPAQ: International physical activity questionnaire

GEE: Generalized estimating equations

LMICs: Low- and Middle-Income Countries

National Commission for Science Technology and Innovation

NCDs: Non-communicable diseases

SSA: Sub Saharan Africa

RCT: Randomized controlled trial

WHO: World Health Organization

# Abstract

In Sub Saharan Africa (SSA) there is a growing burden of Non communicable diseases (NCDs) which poses a big challenge to the weak health system in these resource limited settings. As governments prioritize the control of the COVID 19 pandemic, consequences of chronic diseases such as cardiometabolic risk factors (CMRFs) continue unabated. The aim of this study is to explore innovative strategies to improve blood pressure and body composition through community health workers lifestyle interventions among hypertensive patients in low income populations of Kiambu County, Kenya.

This will be a community-based study which will begin with home-based blood pressure(BP) screening by community health workers (CHW). Those identified as hypertensive will then be recruited to a randomized control trial. The randomized controlled trial (RCT) will involve 52 hypertensive patients willing to be followed up for 6 months. The patient will be randomised to either CHW led lifestyle intervention or usual care arm. The intervention will be implemented for 6 months with outcome assessment at 3 and 6 months. The recruitment will be conducted in level 3 primary health care facilities in Juja and Ruiru Sub-counties in Kiambu County.

An adapted WHO stepwise questionnaire will be used to collect information on demographic characteristics, history of hypertension, other cardiovascular disease (CVD) risk factors, and health behaviours participants at baseline and follow-up visits. The international physical activity questionnaire (IPAQ) will be used to collect data on physical activity. To assess the outcome of the intervention survey will be conducted at baseline, 3 months and 6 months. The research assistants will be blinded to the randomization status of the participants. We will use STATA version 15 software for statistical analyses. The differences between the control and intervention groups on baseline demographics, biological and behavioral measures will be assessed using nonparametric (Wilcoxon rank-sum test, chi square test) and parametric tests(t-test) as appropriate. Generalized estimating equations (GEE) approach will be used to analyze how BP vary over the six months after adjusting for intervention group. This study will not only provide an innovative approach to the management of hypertension through CHW primary health prevention but will also provide preliminary data on feasibility and acceptability of integrating CHW in the control of hypertension for a future fully- powered RCT.

# BACKGROUND INFORMATION

Globally, the COVID-19 pandemic is taking an enormous toll on public health with the priority being to control the pandemic. As governments prioritize the control of the pandemic, consequences of chronic diseases such as cardiometabolic risk factors (CMRFs) continue unabated (Brook et al., 2020). Studies have documented a high mortality and morbidity from other causes as an indirect effect of COVID-19 pandemic resulting from the lack of routine preventative care, delays in diagnosis of new diseases and disruptions to treatment of chronic conditions.

Cardiometabolic risk factors account for the largest proportion of global mortality(Yusuf et al., 2020). The proportion of controlled hypertension is also low, especially in Low and Middle Income Countries (LMICs) with only 7.7% of patients with hypertension having controlled blood pressure (BP) (<140/90 mmHg) in LMICs(Mills et al., 2016). In Sub Saharan Africa (SSA) there is a growing burden of Non communicable diseases (NCDs) which poses a big challenge to the weak health system in these resource limited settings(Moucheraud, 2018). Further, management of cardiovascular diseases (CVDs) in low resource settings is challenging due to limited human and financial resources. There is a need for research to explore innovative, cost effective, and contextually relevant interventions to control BP levels and other cardiovascular risks factors as well as the associated morbidity and mortality in LMICs("Poster Abstracts from the 7th Annual CUGH Conference: Bridging to a Sustainable Future in Global Health," 2016) even in the midst of COVID-19 pandemic.

The dual burden of infectious and NCDs in SSA requires innovative and sustained investment in primary health interventions. Community health workers (CHWs) are a low-cost integral part of health-care delivery system in Kenya and other LMICs. With the shortage of health workers, CHWs have the potential to supplement the formal health system in the struggle to achieve universal health coverage in the era of dual burden of infectious diseases and NCDs in Kenya and other LMICs. Community health workers have been used effectively in the developed world for the management and control of hypertension(Perry et al., 2014). CHW may remove barriers to BP control and medication adherence due to cultural, educational, and language differences between community members and the health care system(Brownstein et al., 2005). A systematic review on the effectiveness of CHW interventions for management of hypertension in the United States showed significant improvement in BP control particularly among the poor, urban minority communities(Brownstein et al., 2007). Similarly, community health workers home-based interventions have shown positive impact in the management and control of hypertension in LMICs (Cappuccio et al., 2006; He et al., 2017; T. Jafar et al., 2009; Neupane et al., 2018; Vedanthan et al., 2019).

One of the ways to address the emerging burden of hypertension could be through home BP monitoring and lifestyle interventions led by CHW. Home BP monitoring has been proven as an effective tool in the management of hypertension(Glynn et al., 2010; Pickering et al., 2008). Compared to measurements in a health facility, home BP monitoring minimizes the “white coat” effect and allows for frequent and multiple readings(Stergiou & Bliziotis, 2011; Verberk et al., 2005) which provides additional BP measurements leading to better treatment decisions. It has been shown that use of electronic cuffs for home BP monitoring is more effective in reaching target BP reduction goals than BP monitoring in a clinical setting alone (Cappuccio et al., 2004) and when combined with other intervention such as patients education and lifestyle counselling is associated with even better BP control(Tucker et al., 2017).

**1.1 Problem statement**

Hypertension is the leading risk factor for deaths due to CVDs and as such, the World Health Organization (WHO) targets to have a worldwide 25% reduction in the prevalence of hypertension by the year 2025 (World Health Organization, 2014). The STEPwise survey shows 24% of Kenyans either had elevated BP or were on treatment for hypertension. Only 8% of the hypertensive persons were on treatment, and among them, only 4.6% had controlled BP (Kenya Ministry of Health, 2016).

Based on literature, modi­fication of lifestyle factors can delay onset of hypertension and can contribute to lowering of BP in treated patients (Gabb et al., 2016; Whelton et al., 2002). Systematic reviews have shown efficacy of interventions focused on physical inactivity (Pescatello et al., 2019; Smart et al., 2020) and dietary approaches to stop hypertension (DASH) (Filippou et al., 2020; Saneei et al., 2014; Siervo et al., 2015) in lowering BP among adults with or without hypertension in different settings. However, we do not know the acceptability of these behavioural interventions in the control of hypertension in Kenya. Clinical trials have also demonstrated the effectiveness of community based CHW interventions in lowering BP among both hypertensive patients(He et al., 2017; Jafar et al., 2010) as well as non-hypertensive population(T. H. Jafar et al., 2009; Neupane et al., 2018) in resource limited settings.

CHWs are an affordable and sustainable solution for behavioural intervention delivery, and an important linkage between community and health care system(Gilmore & McAuliffe, 2013). In light of critical shortages in the health workforce in LMICs, CHWs defined as members of a community with minimal formal training on health problems who provide basic health and medical care to their community, are increasingly recognized as an essential part of the health workforce needed to achieve public health goals (Jeet et al., 2017; Kok et al., 2015; Woldie et al., 2018; World Health Organization). However, the acceptability and effectiveness of CHW primary health intervention in the control of hypertension in Kenya has not been explored. Previous research conducted in Western Kenya demonstrated efficacy of CHW intervention in improving linkage to hypertension health care (Vedanthan et al., 2019) in the general population. However, the feasibility and acceptability of community based CHW lifestyle modification interventions in reduction of blood pressure in Kenya is not known. There is a need for further studies to support primary health interventions geared towards control of hypertension and CVD outcomes among hypertensive patients in the Nairobi metropolitan area. The current study proposes to test the acceptability and efficacy of a CHW-led lifestyle homebased intervention for BP reduction among hypertensive patients.

## 1.2 Significance

This study will not only provide an innovative approach to the management of hypertension through CHW primary health prevention but will also provide preliminary data on feasibility and acceptability of integrating CHW in the control of hypertension for a future fully- powered RCT. CHW have been used effectively in LMICs to deliver NCDs preventive services, using informational as well as behavioural approaches with focus on primordial prevention of NCDs and screening as part of early diagnosis and management. However, there is a lack of uptake of evidence-based community based CHW interventions for reduction of blood pressure in the Kenyan settings. While previous CHW hypertension interventions in LMIC have focused on screening and health education, the current study proposes to adapt a multicomponent evidence-based lifestyle intervention that incorporates behaviour communication and practical individualized lifestyle interventions to reduce BP and other CVD risks among low income population in Kiambu county.

## 1.3 Conceptual framework

# 2.0 RESEARCH QUESTIONS AND STUDY OBJECTIVES

## 2.1 Research Questions

The aim of this study is to explore innovative strategies to improve blood pressure and body composition through CHW lifestyle interventions for hypertensive patients in low income populations of Kiambu County, Kenya. Specific research questions are;

1. What is the prevalence of hypertension among adults in selected wards in Kiambu County?
2. What is the baseline cardio-metabolic risk among hypertensive patients in Kiambu County?
3. What is the effectiveness of a multicomponent CHW lifestyle intervention for control of BP among hypertensive patients in Kiambu County?
4. What is the acceptability of a multicomponent CHW lifestyle intervention for control of blood pressure among hypertensive patients in Kiambu County?

## 2.2 Broad Objectives:

To determine the feasibility and preliminary efficacy of a homebased intervention for the control and management of hypertension amidst the COVID-19 Pandemic in Kiambu County, Kenya

## 2.3 Specific Objectives

i. To determine the prevalence of hypertension among adults in selected wards in Kiambu County.

ii. To determine the baseline cardio-metabolic risk among hypertensive patients in Kiambu County.

iii. To determine the effectiveness of a multicomponent CHW lifestyle intervention for control of BP among hypertensive patients in Kiambu County.

iv. To determine the acceptability of a multicomponent CHW lifestyle intervention for control of blood pressure among hypertensive patients in Kiambu County.

# METHODOLOGY

3.1 Research design

This will be a randomized controlled trial (RCT). Hypertensive patient will be randomised to either CHW led lifestyle intervention or usual care arm. The intervention will be implemented for 6 months with outcome assessment at 3 and 6 months.

3.2 Study area

The study will be conducted in level 3 primary health care facilities in Juja and Ruiru Sub-counties in Kiambu County.

3.3 Study population

Patients with uncontrolled BP (systolic ≥140 mmHg and/or diastolic ≥90 mmHg measured on at least 2 separate screening measurements), and willing to be followed up for 6 months.

### 3.3.1 Inclusion criteria

Hypertensive individuals aged 18 years and above, receiving primary care from the participating Juja and Ruiru sub-counties.

### 3.3.2 Exclusion criteria

Elderly hypertensive patients aged above 70 years and hypertensive patients who wish to relocate from the study area during the study period will be excluded.

## 3.4 Sample size and power calculation

With changes in BP as the primary outcome we calculated a sample of 52 between the 2 groups to show changes in BP between the intervention and control groups at a significance level of 0.05 for a two-sided test with 80% statistical power of finding a large effect (*Cohen’s d*=0.80). This study will be underpowered to find significantly small effect (*Cohen’s d*=0.30) and moderate effect (*Cohen’s d*=0.50), however, we will be able to calculate an effect size and the direction of its impact.

## 3.5 Sampling and participants recruitment

We will randomly sample 2 wards in Ruiru and Juja Sub-counties. All participants in the selected wards with uncontrolled BP willing to participate will be recruited into the study. Screening will be done at the community level. CHW will conduct door to door screening for hypertension using digital BP machines for all adults in the selected wards. The CHW will document the BP readings and anthropometric measurement in a paper form register. The CHW will offer a referral letter (to the nearest level 3 hospital) for all participants with uncontrolled BP. The CHW will furnish the study coordinator with the particulars of such participants for follow-up and possible recruitment into the study. Participants will receive a refund of the transport cost upon presenting the referral note so as to encourage them to turn up at the health facility. Participants who do not show up in the facility within 48 hours of screening will be traced through home visits. The participants will be recruited for the RCT at the level 3 health facility in the respective wards after reassessment by the clinician and commencement of medical management.

## 3.6 Data collection

An adapted WHO stepwise questionnaire will be used to collect information on demographic characteristics (age, sex, occupation), history of hypertension, other CVD risk factors, and health behaviours (smoking, alcohol drinking, diet, and physical activity) of participants at baseline and follow-up visits. The international physical activity questionnaire (IPAQ)(Craig et al., 2003) will be used to collect data on physical activity. The tools will be translated and validated in the local context. We will collect detailed data on patients care cascade during the home visits and monitor their ability to control their BP with the combination of medical management and the CHW intervention. Three BP measurements, body weight, height and waist circumference will be obtained at each data collection visit by outcome assessors masked to intervention assignment.

The primary outcomes will be the differences in systolic and diastolic BP changes from baseline to the end line survey. Secondary outcomes will include the proportion of patients with controlled hypertension (BP <140/90 mm Hg) ) according to the Kenyan national guidelines(Division of Non-Communicable Diseases-Ministry of Health, 2018), body composition – Body mass index(BMI), waist circumference . Low physical activity will be defined as less than 150 minutes of moderate physical activity or 75 minutes of vigorous activity each week. To assess the outcome of the intervention the survey conducted at baseline will be conducted at 3 months of follow-up and at the end of 6 months of intervention.

## 3.7 Data for feasibility and acceptability assessment

At the end of the study, participants in the intervention arm will complete the Client Satisfaction Questionnaire(Larsen et al., 1979) to assess acceptability of the CHW intervention. In addition, 10 patients (purposively sampled to include both gender and different age groups) and CHWs from the intervention group will be targeted for in-depth interviews (IDIs) to understand their experiences and perceptions on the utility of the intervention in the control of hypertension. The IDI guide will cover questions on experiences from using the intervention and determinants of its use. We will assess feasibility of the intervention by examining recruitment rates, follow-up home visits completion rate, dropouts, and adherence to the individualized physical activity program.

## 3.8 Study procedure

The study coordinator and the research assistants will complete a baseline assessment for the illegible participants. This will involve a survey to collect sociodemographic variables, measuring BP, anthropometric measurements (Height, weight and waist circumference) and lifestyle behaviour. Patients with uncontrolled blood pressure will be randomized either control arm (usual care) or intervention arm (Figure 1). The intervention will include a multicomponent community health worker–led home intervention (health coaching, home BP monitoring, BP audit and feedback, physical activity program to be implemented over a period of six (6) months. Participants randomized to the intervention will receives 2 home visits by the trained CHW over a period of 1 month, followed by monthly follow-up visits for a period of 6 months. The initial visit will involve a 90-minute home visit to discuss general knowledge about hypertension and offer tailored counselling on lifestyle modification as well as set physical activity targets. Subsequent monthly visit will be focused on social support, goal setting, problem solving, and maintaining motivation to meet set targets. The visits will involve BP monitoring during which the client and CHW will evaluate the BP and physical activity targets.

To assess the outcome of the intervention the survey conducted at baseline will be conducted by two trained research assistants (who were not part of and have no relationship with the CHWs team) to evaluate the participants at 1) 3 months and 2) 6 months when the intervention has ended. The research assistants will be blinded to the randomization status of the participants. The home visits/clinic visit for evaluation will be scheduled during the first half of the day to minimize the effect of diurnal variations in BP.

Materials to be used will include automatic BP machine, tape measure, a bathroom weighing scale, smart watch fitness monitor and a smart phone with intervention protocol detailing the health education on lifestyle intervention for reducing BP. A paper-based protocol will also be provided for back-up.

## 3.9 Data analysis

We will use STATA version 15 software for statistical analyses. The differences between the control and intervention groups on baseline demographics, biological and behavioral measures will be assessed using nonparametric (chi square test) and parametric tests(t-test) as appropriate. Generalized estimating equations (GEE) approach of Zegar and Liang(Zeger & Liang, 1986) will be used to analyze how BP vary over the six months after adjusting for intervention group, baseline characteristics and healthy diet while adjusting for demographic characteristics. For the secondary outcome variables, we will use GEE analysis for changes in BMI and mixed model analysis of changes in WHtR after adjusting for the interventions ,baseline characteritics and use of antihypertensives. The GEE model will be used to examine the association between the CHW intervention with BP control and normal BMI, reporting odds ratios, after controlling for baseline measurements and use of antihypertensive medication. Statistical significance will be considered at P < 0.05.

##

## 3.10 Ethical considerations

The study will seek ethical approval from JKUAT Institutional ethics committee. Research permit will also be sought from the National Commission of Science Technology and Innovation. Confidentiality and anonymity of patients will be guaranteed by excluding unique identifiers from the data collected from participants. Participation in the study will be on voluntary basis and informed consent will be obtained from the patients and Community health workers (who will be respondents in the IDIs) before data collection. Results of the study will be disseminated through feedback to the participating health facilities and county department of health, conference abstract and peer referred journals.

## 3.11 Project Timeframe

| **Activity/Time** | **Month 0** | **Month 1-3** | **Month 4-6** | **Month 7-9** | **Month 10-12** |
| --- | --- | --- | --- | --- | --- |
| Ethical approval | ***** |  |  |  |  |
| Designing the intervention. |  |  |  |  |  |
| Training of CHV and research assistants |  |  |  |  |  |
| Screening and Recruitment of participants and Baseline assessments. |  |  |  |  |  |
| Baseline data analysis |  |  |  |  |  |
| Intervention and follow-up |  |  |  |  |  |
| End line data collection |  |  |  | **Month 9** |  |
| Acceptability qualitative data collection |  |  |  |  | **Month 10** |
| Data analysis and manuscript Writing |  |  |  |  |  |
| Dissemination of results  *(community, health workers and county department of health, conference, seminars, manuscript)* |  |  |  |  |  |

# REFERENCES

Brook, R. D., Levy, P., & Rajagopalan, S. (2020). Cardiometabolic risk factor control during times of crises and beyond. *Circulation: cardiovascular quality and outcomes*, *13*(7), e006815.

Brownstein, J. N., Bone, L. R., Dennison, C. R., Hill, M. N., Kim, M. T., & Levine, D. M. (2005). Community health workers as interventionists in the prevention and control of heart disease and stroke. *Am J Prev Med*, *29*(5), 128-133.

Brownstein, J. N., Chowdhury, F. M., Norris, S. L., Horsley, T., Jack, L., Jr., Zhang, X., & Satterfield, D. (2007). Effectiveness of community health workers in the care of people with hypertension. *Am J Prev Med*, *32*(5), 435-447. <https://doi.org/10.1016/j.amepre.2007.01.011>

Cappuccio, F. P., Kerry, S. M., Forbes, L., & Donald, A. (2004). Blood pressure control by home monitoring: meta-analysis of randomised trials. *Bmj*, *329*(7458), 145.

Cappuccio, F. P., Kerry, S. M., Micah, F. B., Plange-Rhule, J., & Eastwood, J. B. (2006). A community programme to reduce salt intake and blood pressure in Ghana [ISRCTN88789643]. *BMC Public Health*, *6*(1), 13.

Craig, C. L., Marshall, A. L., Sjöström, M., Bauman, A. E., Booth, M. L., Ainsworth, B. E., Pratt, M., Ekelund, U., Yngve, A., Sallis, J. F., & Oja, P. (2003). International physical activity questionnaire: 12-country reliability and validity. *Med Sci Sports Exerc*, *35*(8), 1381-1395. <https://doi.org/10.1249/01.Mss.0000078924.61453.Fb>

Division of Non-Communicable Diseases-Ministry of Health. (2018). *Kenya National Guidelines for Cardiovascular Diseases Management.*

Filippou, C. D., Tsioufis, C. P., Thomopoulos, C. G., Mihas, C. C., Dimitriadis, K. S., Sotiropoulou, L. I., Chrysochoou, C. A., Nihoyannopoulos, P. I., & Tousoulis, D. M. (2020). Dietary Approaches to Stop Hypertension (DASH) Diet and Blood Pressure Reduction in Adults with and without Hypertension: A Systematic Review and Meta-Analysis of Randomized Controlled Trials. *Adv Nutr*. <https://doi.org/10.1093/advances/nmaa041>

Gabb, G. M., Mangoni, A. A., Anderson, C. S., Cowley, D., Dowden, J. S., Golledge, J., Hankey, G. J., Howes, F. S., Leckie, L., Perkovic, V., Schlaich, M., Zwar, N. A., Medley, T. L., & Arnolda, L. (2016). Guideline for the diagnosis and management of hypertension in adults - 2016. *Med J Aust*, *205*(2), 85-89. <https://doi.org/10.5694/mja16.00526>

Gilmore, B., & McAuliffe, E. (2013). Effectiveness of community health workers delivering preventive interventions for maternal and child health in low-and middle-income countries: a systematic review. *BMC Public Health*, *13*(1), 847.

Glynn, L. G., Murphy, A. W., Smith, S. M., Schroeder, K., & Fahey, T. (2010). Self-monitoring and other non-pharmacological interventions to improve the management of hypertension in primary care: a systematic review. *British Journal of General Practice*, *60*(581), e476-e488.

He, J., Irazola, V., Mills, K. T., Poggio, R., Beratarrechea, A., Dolan, J., Chen, C.-S., Gibbons, L., Krousel-Wood, M., Bazzano, L. A., Nejamis, A., Gulayin, P., Santero, M., Augustovski, F., Chen, J., Rubinstein, A., & Investigators, H. (2017). Effect of a Community Health Worker-Led Multicomponent Intervention on Blood Pressure Control in Low-Income Patients in Argentina: A Randomized Clinical Trial. *Jama*, *318*(11), 1016-1025. <https://doi.org/10.1001/jama.2017.11358>

Jafar, T., Hatcher, J., Poulter, N., Islam, M., Hashmi, S., Qadri, Z., Bux, R., Khan, A., Jafary, F., & Hameed, A. (2009). Hypertension Research Group: Community-based interventions to promote blood pressure control in a developing country: a cluster randomized trial. *Ann Intern Med*, *151*(9), 593-601.

Jafar, T. H., Hatcher, J., Poulter, N., Islam, M., Hashmi, S., Qadri, Z., Bux, R., Khan, A., Jafary, F. H., Hameed, A., Khan, A., Badruddin, S. H., & Chaturvedi, N. (2009). Community-based interventions to promote blood pressure control in a developing country: a cluster randomized trial. *Ann Intern Med*, *151*(9), 593-601. <https://doi.org/10.7326/0003-4819-151-9-200911030-00004>

Jafar, T. H., Islam, M., Hatcher, J., Hashmi, S., Bux, R., Khan, A., Poulter, N., Badruddin, S., & Chaturvedi, N. (2010). Community based lifestyle intervention for blood pressure reduction in children and young adults in developing country: cluster randomised controlled trial. *Bmj*, *340*, c2641. <https://doi.org/10.1136/bmj.c2641>

Jeet, G., Thakur, J. S., Prinja, S., & Singh, M. (2017). Community health workers for non-communicable diseases prevention and control in developing countries: Evidence and implications. *PLoS One*, *12*(7), e0180640-e0180640. <https://doi.org/10.1371/journal.pone.0180640>

Kenya Ministry of Health. (2016). *Kenya STEPwise Survey for Non Communicable Diseases Risk Factors 2015 report . Nairobi: Ministry of Health, Division of Non-CommunicableDiseases.* Retrieved 11th March from <http://www.health.go.ke/wp-content/uploads/2016/04/Steps-Report-NCD-2015.pdf>

Kok, M. C., Dieleman, M., Taegtmeyer, M., Broerse, J. E., Kane, S. S., Ormel, H., Tijm, M. M., & de Koning, K. A. (2015). Which intervention design factors influence performance of community health workers in low-and middle-income countries? A systematic review. *Health Policy Plan*, *30*(9), 1207-1227.

Larsen, D. L., Attkisson, C. C., Hargreaves, W. A., & Nguyen, T. D. (1979). Assessment of client/patient satisfaction: development of a general scale. *Evaluation and program planning*, *2*(3), 197-207.

Mills, K. T., Bundy, J. D., Kelly, T. N., Reed, J. E., Kearney, P. M., Reynolds, K., Chen, J., & He, J. (2016). Global disparities of hypertension prevalence and control: a systematic analysis of population-based studies from 90 countries. *Circulation*, *134*(6), 441-450.

Moucheraud, C. (2018). Service readiness for noncommunicable diseases was low in five countries in 2013–15. *Health Affairs*, *37*(8), 1321-1330.

Neupane, D., McLachlan, C. S., Mishra, S. R., Olsen, M. H., Perry, H. B., Karki, A., & Kallestrup, P. (2018). Effectiveness of a lifestyle intervention led by female community health volunteers versus usual care in blood pressure reduction (COBIN): an open-label, cluster-randomised trial. *The Lancet Global Health*, *6*(1), e66-e73.

Perry, H. B., Zulliger, R., & Rogers, M. M. (2014). Community health workers in low-, middle-, and high-income countries: an overview of their history, recent evolution, and current effectiveness. *Annual review of public health*, *35*, 399-421.

Pescatello, L. S., Buchner, D. M., Jakicic, J. M., Powell, K. E., Kraus, W. E., Bloodgood, B., Campbell, W. W., Dietz, S., DiPietro, L., & George, S. M. (2019). Physical activity to prevent and treat hypertension: A systematic review. *Medicine & Science in Sports & Exercise*, *51*(6), 1314-1323.

Pickering, T. G., Miller, N. H., Ogedegbe, G., Krakoff, L. R., Artinian, N. T., & Goff, D. (2008). Call to action on use and reimbursement for home blood pressure monitoring: a joint scientific statement from the American Heart Association, American Society of Hypertension, and Preventive Cardiovascular Nurses Association. *Hypertension*, *52*(1), 10-29.

Poster Abstracts from the 7th Annual CUGH Conference: Bridging to a Sustainable Future in Global Health. (2016). [Conference Review]. *Annals of global health*, *82*(3). <https://www.embase.com/search/results?subaction=viewrecord&id=L614045063&from=export>

Saneei, P., Salehi-Abargouei, A., Esmaillzadeh, A., & Azadbakht, L. (2014). Influence of Dietary Approaches to Stop Hypertension (DASH) diet on blood pressure: a systematic review and meta-analysis on randomized controlled trials. *Nutrition, Metabolism and Cardiovascular Diseases*, *24*(12), 1253-1261.

Siervo, M., Lara, J., Chowdhury, S., Ashor, A., Oggioni, C., & Mathers, J. C. (2015). Effects of the Dietary Approach to Stop Hypertension (DASH) diet on cardiovascular risk factors: a systematic review and meta-analysis. *British Journal of Nutrition*, *113*(1), 1-15.

Smart, N. A., Howden, R., Cornelissen, V., Brook, R., McGowan, C., Millar, P. J., Ritti-Dias, R., Baross, A., Carlson, D. J., & Wiles, J. D. (2020). Physical Activity to Prevent and Treat Hypertension: A Systematic Review. *Medicine and Science in Sports and Exercise*, *52*(4), 1001-1002.

Stergiou, G. S., & Bliziotis, I. A. (2011). Home blood pressure monitoring in the diagnosis and treatment of hypertension: a systematic review. *American journal of hypertension*, *24*(2), 123-134.

Tucker, K. L., Sheppard, J. P., Stevens, R., Bosworth, H. B., Bove, A., Bray, E. P., Earle, K., George, J., Godwin, M., & Green, B. B. (2017). Self-monitoring of blood pressure in hypertension: a systematic review and individual patient data meta-analysis. *PLoS Med*, *14*(9), e1002389.

Vedanthan, R., Kamano, J. H., DeLong, A. K., Naanyu, V., Binanay, C. A., Bloomfield, G. S., Chrysanthopoulou, S. A., Finkelstein, E. A., Hogan, J. W., & Horowitz, C. R. (2019). Community health workers improve linkage to hypertension care in western Kenya. *Journal of the American College of Cardiology*, *74*(15), 1897-1906.

Verberk, W. J., Kroon, A. A., Kessels, A. G., & de Leeuw, P. W. (2005). Home blood pressure measurement: a systematic review. *Journal of the American College of Cardiology*, *46*(5), 743-751.

Whelton, S. P., Chin, A., Xin, X., & He, J. (2002). Effect of aerobic exercise on blood pressure: a meta-analysis of randomized, controlled trials. *Ann Intern Med*, *136*(7), 493-503.

Woldie, M., Feyissa, G. T., Admasu, B., Hassen, K., Mitchell, K., Mayhew, S., McKee, M., & Balabanova, D. (2018). Community health volunteers could help improve access to and use of essential health services by communities in LMICs: an umbrella review. *Health Policy Plan*, *33*(10), 1128-1143.

World Health Organization. Global Health Workforce Alliance. Global experience of community health workers for delivery of health related millennium development goals: a systematic review, country case studies, and recommendations for integration into national health systems. 2010 [cited 2011 Mar 22]. In.

World Health Organization. (2014). *Global status report on noncommunicable diseases 2014*. World Health Organization.

Yusuf, S., Joseph, P., Rangarajan, S., Islam, S., Mente, A., Hystad, P., Brauer, M., Kutty, V. R., Gupta, R., & Wielgosz, A. (2020). Modifiable risk factors, cardiovascular disease, and mortality in 155 722 individuals from 21 high-income, middle-income, and low-income countries (PURE): a prospective cohort study. *The Lancet*, *395*(10226), 795-808.

Zeger, S. L., & Liang, K.-Y. (1986). Longitudinal data analysis for discrete and continuous outcomes. *Biometrics*, 121-130.

# APPENDIX 2: SURVEY QUESTIONNAIRE

## An adapted WHO stepwise questionnaire to collect prevalence of cardiometabolic risk factors among low income population in Kiambu County.

|  | Phone Number |  |
| --- | --- | --- |
|  | **Demographic Data** | |
|  | Age |  |
|  | Gender | 1. Male 2. Female |
|  | Highest Level Of Education | 1.Never been to school  2.Primary  3.Secondary  4.University/college  5.Other (specify |
|  | Marital status | 1.Single  2.Married  3.Divorced/separated/widowed  4.other (specify) |
|  | Work status over the past 12 months | 1. Employed  2. Self-employed  3. Homemaker  4. Retired  5. Farmer  6.Student  7. Others |
|  | Average earnings of the household for the past 1 year | ---------------------- per day  ----------------------per week  ---------------------per month |
|  | **Behavioural Measurements** | |
|  | Use of Tobacco |  |
|  | Do you use any **tobacco products** i..e cigarettes, cigars or pipes? | 1YES  2 NO |
|  | How often do you smoke cigarette? |  |
|  | At what age did you start smoking cigarette daily? |  |
|  | At what age did you **quit** smoking **daily**? |  |
|  | Do you **use** any **smokeless tobacco** *[snuff, chewing tobacco, betel]*? | 1.YES  2.NO |
|  | How often do you **use smokeless tobacco** |  |
|  | In the past 1 week has someone **in your home** smoked in your presence? | 1. YES 2. NO |
|  | In the past 1 week has someone smoked in closed areas **in your workplace** in your presence? | 1.YES  2.NO |
|  | **Alcohol Consumption** | |
|  | Have you **ever** consumed an alcohol such as beer, wine, spirits, busaa, changaa? | 1.YES  2.NO |
|  | In the past year, have you consumed alcohol? | 1.YES  2.NO |
|  | In the past 1 year how often have you consumed alcohol? | 1 Daily  2 5-6 days per week  31-4 days per week  4 1-3 days per month  5 Less than once a month |
|  | Have you taken alcohol in the past 1 month? | 1.YES  2.NO |
|  | How often have you consumed alcohol with meals | Usually with meals  Sometimes with meals  Rarely with meals  Never with meals |
|  | **Diet** | |
|  | How many days do you **eat fruit in week**? |  |
|  | How many days in a week do you **eat vegetables** |  |
|  | What type of  cooking **oil or fat is used in your household?** | 1.Vegetable oil  2.Butter or ghee  3.Margarine  4. None used  5.Other |
|  | How many meals per week do you eat outside your home |  |
|  | **Physical activity ( IPAQ)**  The questions will ask you about the time you spent being physically active in the last 7 days. Please answer each question even if you do not consider yourself to be an active person. Please think about the activities you do at work, as part of your house and yard work, to get from place to place, and in your spare time for recreation, exercise or sport. | |
|  | Think about all the vigorous activities that you did in the last 7 days. Vigorous physical activities refer to activities that take hard physical effort and make you breathe much harder than normal. Think only about those physical activities that you did for at for atleast 10 minutes at a time.  During the last 7 days, on how many days did you do vigorous physical activities like heavy lifting, digging, aerobics, or fast bicycling? | 1. -------------- days per week 2. No vigorous activity (**Skip to number 3)** |
|  | How much time did you usually spend doing vigorous physical activities on one  of those days? | 1. ………… hours per day  2. ………… minutes per day  Don’t know/Not sure |
|  | Think about all the moderate activities that you did in the last 7 days. Moderate activities refer to activities that take moderate physical effort and make you breathe somewhat harder than normal. Think only about those physical activities that you did for at least 10 minutes at a time.  During the last 7 days, on how many days did you do moderate physical activities like carrying light loads, bicycling at a regular pace, or doubles tennis?  Do not include walking. | 1.--------------------days per week  2. No moderate physical activities **(Skip to question 5)** |
|  | How much time did you usually spend doing moderate physical activities on one  of those days? | 1……………hours per day  2……………. minutes per day  3.Don’t know/Not sure |
|  | Think about the time you spent walking in the last 7 days. This includes at work and at home, walking to travel from place to place, and any other walking that you have done solely for recreation, sport, exercise, or leisure.  During the last 7 days, on how many days did you walk for at least 10 minutes  at a time? | 1.……………. days per week  2.No walking Skip to question 7 |
|  | *The last question is about the time you spent sitting on weekdays during the last 7 days. Include time spent at work, at home, while doing course work and during leisure time. This may include time spent sitting at a desk, visiting friends, reading, or sitting or lying down to watch television*.  During the last 7 days, how much time did you spend sitting on a week day? | 1.…………… hours per day  2……………. minutes per day  Don’t know/Not sure |
| **History of Raised Blood Pressure** | | |
|  | Have you ever had your blood pressure measured? |  |
|  | Have you ever been told by a doctor or other health worker that you have raised blood pressure or hypertension? |  |
|  | Are you on any treatments/advice for high blood pressure? | |
|  | Drugs (medication) that you have taken in the past two weeks |  |
|  | Advice to reduce salt intake |  |
|  | Advice or treatment to stop smoking |  |
|  | Advice to start or do more exercise |  |
| **Step 2 Physical Measurements** | | |
|  | Interviewer ID |  |
|  | Device IDs for height and weight |  |
|  | Height(cm) |  |
|  | Weight(kg) |  |
|  | **For women:** Are you pregnant? |  |
|  | Waist circumference(cm) |  |

# APPENDIX 3: CLIENT SATISFACTION QUESTIONNAIRE.

1. What is your take on the use of CHW intervention for managing Blood pressure

Satisfactory ( )

Neutral ( )

Not satisfactory ( )

If satisfactory why

If unsatisfactory, why

1. Would you liketo continue with the CHW lifestyle intervention ?

Yes ( ) No ( )

If no, why

1. Would you recommend that other patients be supported by CHW in the management of blood pressure through lifestyle intervention?

Yes ( ) No ( )

If no, why

1. Do you have any complain(s) in regard to this method of being supported by CHW in the management of blood pressure through lifestyle intervention?

Yes ( ) No ( )

If yes:-

What were the complains

1. Do you any commendation(s) in regard to this method of being supported by CHW in the management of blood pressure through lifestyle intervention?

Yes ( ) No ( )

If yes: -

1. What were the commendations
2. What was the reaction of most of the other family members towards you being supported by CHW in the management of blood pressure through lifestyle intervention?

Positive( )

Negative ( )

1. What was the reaction of most of the other community members about hypertensive patients supported by CHW in the management of blood pressure through lifestyle intervention?

Positive ( )

Negative ( )
