# Supplementary material for: Preliminary efficacy of a community health worker homebased intervention for the control and management of hypertension in Kiambu County, Kenya- a randomized control trial
Source: PLoS One. 2024 Aug 29;19(8):e0293791. doi: 10.1371/journal.pone.0293791 (PMC11361652; doi:10.1371/journal.pone.0293791)
Supplement: S4 File — (PDF) [file pone.0293791.s005.pdf]

JOMO KENYATTA UNIVERSITY OF AGRICULTURE AND TECHNOLOGY
P.O BOX 62000(00200) NAIROBI, Tel:(067) 58700001-4
(Office of the Deputy Vice Chancellor, Research Production and Extension Division)

JKUAT INSTITUTIONAL SCIENTIFIC AND ETHICAL REVIEW COMMITTEE (ISERC)

REF: JKU/2/4/896B

Date: 30th June 2022

DR GRACE MBUTHIA
SCHOOL OF NURSING, JKUAT

Dear Dr Mbuthia,

RE: FEASIBILITY AND PRELIMINARY EFFICACY OF A HOMEBASED INTERVENTION FOR THE CONTROL AND MANAGEMENT OF HYPERTENSION AMIDST THE COVID-19 PANDEMIC IN KIAMBU COUNTY, KENYA

This is to inform you that JKUAT Institutional Scientific and Ethical Review Committee (ISERC) has reviewed and approved your above research proposal. Your application approval number is **JKU/IERC/02316/0652.** The approval period is **30th June 2022** to **29th June 2023**.

This approval is subject to compliance with the following requirements;

i. Only approved documents including (informed consents, study instruments, Material Transfer Agreements) will be used
ii. All changes including (amendments, deviations, and violations) are submitted for review and approval by JKUAT ISERC.
iii. Death and life threatening problems and serious adverse events or unexpected adverse events whether related or unrelated to the study must be reported to JKUAT ISERC within 72 hours of notification
iv. Any changes, anticipated or otherwise that may increase the risks or affected safety or welfare of study participants and others or affect the integrity of the research must be reported to JKUAT ISERC within 72 hours
v. Clearance for export of biological specimens must be obtained from relevant institutions.
vi. Submission of a request for renewal of approval at least 60 days prior to expiry of the approval period. Attach a comprehensive progress report to support the renewal.
vii. Submission of an executive summary report within 90 days upon completion of the study to JKUAT ISERC.

Prior to commencing your study, you will be expected to obtain a research license from National Commission for Science, Technology and Innovation (NACOSTI) https://oris.nacosti.go.ke and also obtain other clearances needed.

Yours sincerely

Dr Patrick Mburugu
Chair, JKUAT ISERC

INSTITUTIONAL ETHICS REVIEW COMMITTEE
30 JUN 2022
APPROVED
P.O. Box 62000-00200 NAIROBI
www.jkuat.ac.ke
JKUAT
