# Supplementary material for: Preliminary efficacy of a community health worker homebased intervention for the control and management of hypertension in Kiambu County, Kenya- a randomized control trial
Source: PLoS One. 2024 Aug 29;19(8):e0293791. doi: 10.1371/journal.pone.0293791 (PMC11361652; doi:10.1371/journal.pone.0293791)
Supplement: Attachment — Submitted filename: PONE-D-23-33530 review.docx [file pone.0293791.s007.docx]

*Preliminary efficacy of a community health worker homebased intervention for the control and management of hypertension in Kiambu County, Kenya- A randomized control trial*

This study is a small pilot randomized trial of a community health worker intervention to deliver lifestyle counseling to people with uncontrolled hypertension identified on community screening in Kiambu County, Kenya. The intervention consisted of lifestyle counseling which focused on dietary changes and exercise recommendations and was delivered via monthly visits by a community health worker. All hypertension care was provided at health facilities and was delivered in the same way for intervention and control participants. The study is well written and makes an important contribution. I do think that more detail is needed on the CHWs themselves and potential mechanisms for effects seen. I also think the choice of cluster randomization is a major limitation and is not adequately accounted for in the analysis or in the discussed limitations of the study. In randomizing only two clinic sites, the sample size is effectively n=2 which limits inferences that can be made

Major

1. The manuscript would benefit from additional detail on the CHW and the behavior change intervention. It would be helpful to include information on the following:
   a. What training did CHWs receive (including the content, duration, and frequency of training)?
   b. How were the CHWs compensated and how did this compare to current standard?
   c. How were CHWs supervised? Was there any quality assessment to assess fidelity of the content being delivered?
   d. This is framed as a behavior change intervention. Was there a behavior change framework used to guide CHW training material development?
   e. How was the lifestyle counseling material developed and/or adapted for contextual relevance? How did CHWs adapt advice based on individual participant needs?
2. It appears that randomization was done at the facility level, rather than the patient level, which would yield a sample size of n=2 (methods, line 151-153). This is a major limitation and should be more clearly written in the methods, along with justification for why cluster randomization was chosen, as opposed to individual randomization for this small pilot study. Additionally, the authors should explain how clustering is addressed in the sample size calculations and the analysis. Cluster level randomization should also be mentioned in the limitations as it introduces several potential confounders of study findings. For example, if clinicians at the intervention facility were more assertive about titrating antihypertensive medications, this could have explained observed differences in blood pressure.
3. Line 150 indicates that participants were allocated to either Juja or Ruiru health facilities. Can the authors elaborate on how the assigned facility was chosen for each participant? Presumably this was by proximity to the participant’s home, but the fact that there were identical numbers of patients in each clinic makes me wonder if clinics were assigned in some way. This needs additional clarity.
4. The results on blood pressure reduction are impressive given that intervention was lifestyle counseling only and there were high baseline levels of physical activity. Presumably the mechanisms of change were driven by dietary changes, medication adherence, or both. Is there any information available regarding initiation of antihypertensive medications? It seems this was assessed at least by self-report in the research team surveys and would be helpful to know if the intervention arm received medications more frequently than control participants.
5. In addition to medications, can the authors present data on any other factors that may have changed as a result of the intervention? The current manuscript provides details on clinical outcomes (SBP/DBP, BMI), but it is unclear what mechanisms these changes were achieved through. Presentation of changes in diet and exercise as assessed in the surveys would be helpful. I recognize that formal mediation analysis is not possible in this small pilot study, but observations of changes in diet or exercise could be hypothesis generating for a larger trial.
6. The authors note differential follow-up by trial arm in the research surveys that were conducted independent of clinical care engagement. This should be mentioned in the limitations section. Differences seen here could be a function of the intervention keeping people more engaged over time (thus would bias results towards the null and true intervention effects may be even greater than the authors observed), or could be due to underlying differences in the populations given the cluster randomization.

Minor:

1. In the abstract and 1^st^ introduction sentence, I suggest using the term “resource limited health systems” instead of “weak health systems” as this centeres the description on the primary challenge of resources rather than critique of the health workers/systems themselves.
2. In the methods, I would suggest describing the context of Kiambu County and the included health facilities to facilitate reader understanding of applicability of their setting. Simple description of the context as urban, peri-urban, etc , along with differences between the communities surrounding the two clinics would be helpful.
3. Figure 1: there is an error in the first box – should read “Eligible n=352 high BP (Systolic ≥140 and/or diastolic ≥90 mmHg)”
4. Figure 1: The exclusion criteria in Figure 1 indicate that participants were excluded if they are already engaged in hypertension care at ‘any health facility’ but the text indicates that participants were excluded if in care at a different health facility (other than Ruiru or Juja). Please clarify the criteria and harmonize language between the text and Figure 1.
5. Table 1: do not add up to column total for some characteristics in the usual care arm (gender, smoking) – were there missing data on these characteristics?
