# Supplementary material for: Preliminary efficacy of a community health worker homebased intervention for the control and management of hypertension in Kiambu County, Kenya- a randomized control trial
Source: PLoS One. 2024 Aug 29;19(8):e0293791. doi: 10.1371/journal.pone.0293791 (PMC11361652; doi:10.1371/journal.pone.0293791)
Supplement: Attachment — Submitted filename: Response to reveiwers.docx [file pone.0293791.s008.docx]

**A rebuttal letter**

**Dear Reviewers and editor,**

Thank you very much for taking time to review our manuscript and for the comments given to improve the work. Below is a point by point response to the comments given.

1. *Please ensure that your manuscript meets PLOS ONE's style requirements, including those for file naming.*

Thank you for this comment we have edited the manuscript to meet the PLOS ONE style requirements.

2. *We note that you have selected “Clinical Trial” as your article type. PLOS ONE requires that all clinical trials are registered in an appropriate registry*

We wish to confirm that the the clinical trial was registered with the Pan African Clinical Trial Registry database NO:PACTR202309530525257.

3. *We note that the grant information you provided in the ‘Funding Information’ and ‘Financial Disclosure’ sections do not match. When you resubmit, please ensure that you provide the correct grant numbers for the awards you received for your study in the ‘Funding Information’ section.*

We have provided correct grant numbers.

4. *Thank you for stating the following financial disclosure: "This research was supported by the Consortium for Advanced Research Training in Africa (CARTA). CARTA is jointly led by the African Population and Health Research Center and the University of the Witwatersrand and funded by the Carnegie Corporation of New York (Grant No. G-19-57145), Sida (Grant No:54100113), Uppsala Monitoring Center, Norwegian Agency for Development Cooperation (Norad), and by the Wellcome Trust [reference no. 107768/Z/15/Z] and the UK Foreign, Commonwealth & Development Office, with support from the Developing Excellence in Leadership, Training and Science in Africa (DELTAS Africa) programme. The statements made and views expressed are solely the responsibility of the authors."*

*Please state what role the funders took in the study. If the funders had no role, please state: "The funders had no role in study design, data collection and analysis, decision to publish, or preparation of the manuscript."*

We have added the statement "The funders had no role in study design, data collection and analysis, decision to publish, or preparation of the manuscript." in the financial disclosure.

*Please include this amended Role of Funder statement in your cover letter; we will change the online submission form on your behalf*.

This is well noted and appreciated.

5. *Please include captions for your Supporting Information files at the end of your manuscript, and update any in-text citations to match accordingly. Please see our Supporting Information guidelines for more information: http://journals.plos.org/plosone/s/supporting-information.*

We have included captions for supporting information file at the end of the manuscript and updated the in-test citations as per the guidelines.

**Reviewer #1 Comments**

*1. The manuscript would benefit from additional detail on the CHW and the behavior change intervention. It would be helpful to include information on the following:
a. What training did CHWs receive (including the content, duration, and frequency of training)?
b. How were the CHWs compensated and how did this compare to current standard?
c. How were CHWs supervised? Was there any quality assessment to assess fidelity of the content being delivered?
d. This is framed as a behavior change intervention. Was there a behavior change framework used to guide CHW training material development?
e. How was the lifestyle counseling material developed and/or adapted for contextual relevance? How did CHWs adapt advice based on individual participant needs?*

Thank you for the comments. Details on the intervention have been added in line 194-202. We have noted that the community health workers were trained to coach patients on lifestyle modification, home BP-monitoring, and medication adherence during a 2-day interactive training session followed by onsite field return demonstrations. The CHW were taught culturally appropriate behavioral change related to hypertension based on the Centers for Disease Control(CDC) CHW training manual for preventing heart disease and stroke . Before the actual intervention, several mock intervention sessions were done and critiqued to ensure CHW ability to deliver intervention content consistently. Additionally, the research assistants (qualified nurse) made monthly supervisory visits and sat in the intervention sessions to ensure that home visits and intervention were delivered effectively.

Concerning adapting the counselling to individual needs we have noted that the CHWs were trained to adapt the counseling based on individual participant needs by emphasizing on actions to be taken based on hypertension risk factors identified in the individual client.

Concerning compensation, the CHW were paid the Ministry of Health recommended monthly stipend for CHW of approximately 60 USD per month as noted in line 213-214.

*2. It appears that randomization was done at the facility level, rather than the patient level, which would yield a sample size of n=2 (methods, line 151-153). This is a major limitation and should be more clearly written in the methods, along with justification for why cluster randomization was chosen, as opposed to individual randomization for this small pilot study. Additionally, the authors should explain how clustering is addressed in the sample size calculations and the analysis. Cluster level randomization should also be mentioned in the limitations as it introduces several potential confounders of study findings. For example, if clinicians at the intervention facility were more assertive about titrating antihypertensive medications, this could have explained observed differences in blood pressure.*

We thank the reviewer for this comment. The two health facilities are government owned, of the same level, have similar staffing norms, and use similar treatment guidelines hence it is plausible to assume that the treatment provided is of the same standard. We used the health facilities as the point of recruitment purely for geographical locations to avoid contaminating the control arm due to possible interactions with the intervention participants if they came from the same neighborhoods. We added this as a possible limitation of the study (Line 334).

*3. Line 150 indicates that participants were allocated to either Juja or Ruiru health facilities. Can the authors elaborate on how the assigned facility was chosen for each participant? Presumably this was by proximity to the participant’s home, but the fact that there were identical numbers of patients in each clinic makes me wonder if clinics were assigned in some way. This needs additional clarity.*

This has been clarified as follows; The 2 primary health facilities of same level and government owned were in 2 different sub counties where home blood pressure screening took place and participants were referred to the appropriate facility in their respective Sub-county. The two facilities were then randomly allocated to the intervention through simple random sampling ( two folded papers bearing the name of the two facilities were tossed and one picked at random became the intervention facility). For the purposes of the pilot RCT only 40 participants were recruited in each facility

*4. The results on blood pressure reduction are impressive given that intervention was lifestyle counseling only and there were high baseline levels of physical activity. Presumably the mechanisms of change were driven by dietary changes, medication adherence, or both. Is there any information available regarding initiation of antihypertensive medications? It seems this was assessed at least by self-report in the research team surveys and would be helpful to know if the intervention arm received medications more frequently than control participants.*

Thank you for the comment. Information regarding initiation of antihypertensive medication was documented and any potential influence of medication use at baseline was controlled by statistical adjustment in the outcome models. The intervention involved counselling on medication adherence, dietary advice, home-BP monitoring and setting physical activity targets. Community health workers made regular visits to the households of the intervention participants and thus the observed blood pressure changes can be attributed both to better adherence to antihypertensive medication and lifestyle interventions. However, the level of adherence to medication and dietary changes were not assessed in the subsequent surveys and the mechanism behind the change in BP was not explored in this pilot study. We have added this as a limitation of the study and recommend the same in fully powered trial.

5. In addition to medications, can the authors present data on any other factors that may have changed as a result of the intervention? The current manuscript provides details on clinical outcomes (SBP/DBP, BMI), but it is unclear what mechanisms these changes were achieved through. Presentation of changes in diet and exercise as assessed in the surveys would be helpful. I recognize that formal mediation analysis is not possible in this small pilot study, but observations of changes in diet or exercise could be hypothesis generating for a larger trial.

We thank the reviewer for this comment. As noted comment number 4 the study did not set out behavioural changes as part of the outcomes in the study and did not study the mechanism behind reduced BP in this pilot. We have added this as a limitation of our study.

*6. The authors note differential follow-up by trial arm in the research surveys that were conducted independent of clinical care engagement. This should be mentioned in the limitations section. Differences seen here could be a function of the intervention keeping people more engaged over time (thus would bias results towards the null and true intervention effects may be even greater than the authors observed), or could be due to underlying differences in the populations given the cluster randomization.*

Thanks for this comment. We agree that the intensive follow up of the intervention participants improved their engagement over time leading to higher changes in blood pressure. We have included this in the limitation section and highlighted the potential underestimation of the intervention effect.

7. *In the abstract and 1st introduction sentence, I suggest using the term “resource limited health systems” instead of “weak health systems” as this centeres the description on the primary challenge of resources rather than critique of the health workers/systems themselves.*

Thank you for the suggestion. The term weak health system has been replaced with resource limited.

*8.* *In the methods, I would suggest describing the context of Kiambu County and the included health facilities to facilitate reader understanding of applicability of their setting. Simple description of the context as urban, peri-urban, etc , along with differences between the communities surrounding the two clinics would be helpful.*

We have added a simple description of the setting.

*9. Figure 1: there is an error in the first box – should read “Eligible n=352 high BP (Systolic ≥140 and/or diastolic ≥90 mmHg)”*

Thank you for highlighting this error, we have deleted the incorrect statement.

*10. Figure 1: The exclusion criteria in Figure 1 indicate that participants were excluded if they are already engaged in hypertension care at ‘any health facility’ but the text indicates that participants were excluded if in care at a different health facility (other than Ruiru or Juja). Please clarify the criteria and harmonize language between the text and Figure 1.*

*5. Table 1: do not add up to column total for some characteristics in the usual care arm (gender, smoking) – were there missing data on these characteristics?*

Thank you for this comment , the omission on the counts for gender and smoking has been corrected.

**Reviewer #2 Comments**

*This manuscript presents data analysis from a randomized control trial (RCT) to compare the effectiveness of the CHW homebased intervention (compared to usual care), for control and management of hypertension, in a Kenyan population. The topic is of importance, the study was registered as a RCT (with a valid number), and was approved by the respective IRB/Ethics Committee. While the study objectives sound interesting, is important, and on target, some shortcomings were observed, in regards to abiding by the CONSORT guidelines for conducting and reporting results of high-quality randomized controlled trials (RCTs). Some other (statistical) comments were also provided.*

*1. Abstract:It's better for the readers, if the Abstract was written in sections, such as Objectives, Methods, Results and Conclusion.*

We have now written the abstract in a structured manner.

*2. Methods :Methods reporting need some work. An orderly manner is suggested, following CONSORT guidelines, without repeating information, such as Trial Design, Participant Eligibility Criteria and settings, Interventions, Outcomes, sample size/power considerations, Interim analysis and stopping rules, Randomization (details on random number generation, allocation concealment, implementation), Blinding issues, etc, should be mentioned. The authors are advised to create separate subsections for each of the possible topics (whichever necessary), and that way produce a very clear write up. They are advised to write it carefully, following nice examples in the manuscript below:* https://www.sciencedirect.com/science/article/pii/S0889540619300010
*Specific comments:*

(*a) For instance, the randomization and allocation concealment should be made very clear (they are NOT the same thing); the trial staff recruiting patients should NOT have the randomization list. Randomization should be prepared by the trial statistician, and he/she would not participate in the recruiting.*

We thank the reviewer for this comment. We used simple random sampling to assign the two health facilities into the intervention and control arms . This random assignment followed the patient recruitment at each of the two facilities after their initial screening and referral by community health workers. We consecutively enrolled the new patients until the required sample sizes for each arm were attained. We have added details of the sampling procedure.

(b) *The manuscript does not provide details on the randomization procedure; it's stated that a simple random was done. More details on that was done would be helpful, like how was the random sequence generated. Furthermore, any reasoning, why a block randomization was not used, which is often recommended to ensure a balance in sample size across groups?*
[*https://www.ncbi.nlm.nih.gov/pmc/articles/PMC2267325/*](https://www.ncbi.nlm.nih.gov/pmc/articles/PMC2267325/)

Thank you for the comment. We recruited participants from two health facilities after their initial screening and referral by community health workers. We did not use block randomization because of the consecutive enrolment of participants into the intervention and control arms. Furthermore, to avoid the contamination of the control arm, we selected two health facilities that serve different neighbourhoods.

*(c) Statistical Analysis: (c1) The "Data Analysis" section should be named "Statistical Analysis".*

Thanks for the suggestion. We have changed the name of the section to “Statistical Analysis”.

*(c2) While assessing baseline characteristics, t-test was used which is heavily based on normality assumptions. Under violations, alternative nonparametric tests, such as Wilcoxon rank sum tests, should be mentioned.*

Thanks for this comment. Prior to using the ttest, we plotted histograms to visualize the shape of the distributions of the BMI and blood pressure readings of patients. It is plausible to assume that BP and BMI measurements are normally distributed (based on the histograms), which justifies the use of the t-test. We acknowledge that non-parametric tests like Wilcoxon rank sum tests would be useful especially where the normal distribution assumption is violated.

*(c3) The GEE technique was used to handle repeated measures; any reasoning, why a mixed model (using random effects) was not used?*

We preferred the GEE model because it does not rely on probability distribution assumption hence not subject to misspecification as opposed to mixed effects model (using random effects) which is heavily reliant on the probability distribution underlying the data [(Hubbard et al. 2010)](https://paperpile.com/c/6vr1DS/wKoV). We have added the justification to the statistical analysis section. However, we have also performed a sensitivity analysis using a mixed effects model (using random effects) and presented our results in supporting information (S3 Table).

*(c4) GEE with logit link was used; it's better to clearly write how the binary (response) variable was coded.*

Thanks for the suggestion. We have added details of how the outcome variable was coded.

*3. Results & Conclusions*:

*(a) The authors should check that any statement of significance should be followed by a p-value in the entire Results section. Otherwise, the Results section look OK; it's pretty straightforward.*

We have ensured that statement of significance in the results have p- values.
 *(b) Conclusions should state that the current findings are ONLY based on the random samples derived from an Iranian population, and should allude to future studies with much larger sample sizes and collected at other geographical areas to confirm the effectiveness of the home-based intervention.*

Thank you for your comment, the statement that these findings are based on a small sample derived from a Kenyan population and the need for future studies with bigger samples and from other geographical regions have been added in the conclusion.

**Reviewer #3 Comments**


1. *The manuscript should be edited for language, as it contains several grammar mistakes.*

We have edited the paper for language and corrected the grammatical errors.

*2. The introduction section is too long. Please rewrite it.*

Thank you for the comment, we shortened the introduction as suggested.

*3. The inclusion and exclusion criteria should be clarified*.

The inclusion criteria has been clarified.

4. The study protocol needs some clarification. The questionnaire filled out at the beginning of the study might have increased the awareness in the control group, have the authors noticed any changed in the lifestyle and behaviour if the control group.

Thanks for the comment. While we agree that the initial questionnaire may have increased awareness in the control group, the baseline survey only involved an assessment of their lifestyles, blood pressure and their anthropometric measurements without health education about the required behavior change. The before-after analyses from the paired t-tests, GEE models and mixed effects models indicate a significant difference in mean BP and BMi between the two groups which implies that the intervention was effective.

*5. As for the people in the intervention group who did not adhere to the plan, was there any difference in the BP changes over time with those following the recommendations.*

Thanks for the comment. We did not assess the medication adherence levels in the subsequent surveys and have indicated this as a limitation of our study.
 *6. There is no information on the changes in the behaviour and lifestyle of the intervention group. It is recommended to add those information and also assess how they have affected the final outcome.*

Thanks for the comment. This was not part of the outcomes set out in the study protocol (S3.File) and therefore we did not collect longitudinal data on the behaviour changes in the subsequent surveys. We have highlighted this as a limitation of our study.

**Reference**

[Hubbard, Alan E., Jennifer Ahern, Nancy L. Fleischer, Mark Van der Laan, Sheri A. Lippman, Nicholas Jewell, Tim Bruckner, and William A. Satariano. 2010. “To GEE or Not to GEE: Comparing Population Average and Mixed Models for Estimating the Associations between Neighborhood Risk Factors and Health.” *Epidemiology* 21 (4): 467–74.](http://paperpile.com/b/6vr1DS/wKoV)
